# Reactive Oxygen Species: Not Omnipresent but Important in Many Locations

**DOI:** 10.3389/fcell.2021.716406

**Published:** 2021-09-07

**Authors:** Marc Herb, Alexander Gluschko, Michael Schramm

**Affiliations:** Institute for Medical Microbiology, Immunology and Hygiene, Cologne, Germany

**Keywords:** mitochondria, NADPH oxidases, ROS sources, reactive oxygen species, redox balance, oxidative stress, ROS probes, ROS inhibitors

## Abstract

Reactive oxygen species (ROS), such as the superoxide anion or hydrogen peroxide, have been established over decades of research as, on the one hand, important and versatile molecules involved in a plethora of homeostatic processes and, on the other hand, as inducers of damage, pathologies and diseases. Which effects ROS induce, strongly depends on the cell type and the source, amount, duration and location of ROS production. Similar to cellular pH and calcium levels, which are both strictly regulated and only altered by the cell when necessary, the redox balance of the cell is also tightly regulated, not only on the level of the whole cell but in every cellular compartment. However, a still widespread view present in the scientific community is that the location of ROS production is of no major importance and that ROS randomly diffuse from their cellular source of production throughout the whole cell and hit their redox-sensitive targets when passing by. Yet, evidence is growing that cells regulate ROS production and therefore their redox balance by strictly controlling ROS source activation as well as localization, amount and duration of ROS production. Hopefully, future studies in the field of redox biology will consider these factors and analyze cellular ROS more specifically in order to revise the view of ROS as freely flowing through the cell.

## Reactive Oxygen Species: Handle With Care!

Reactive oxygen species (ROS) are molecules with higher reactivity than molecular oxygen (O_2_) (Fenton, 1894; Haber et al., 1997; D’Autreaux and Toledano, 2007; Hatz et al., 2007; Prousek, 2007; Das and Roychoudhury, 2014; Odobasic et al., 2016; Aratani, 2018; Juan et al., 2021) and include highly reactive free radicals, such as the superoxide anion (O_2_^–^) (Hayyan et al., 2016), and non-radical species, such as hydrogen peroxide (H_2_O_2_) (Chavez et al., 2009; Odobasic et al., 2016; Aratani, 2018).

A plethora of different ROS sources, such as NADPH oxidases (Nox) (Bedard and Krause, 2007; Nakano et al., 2008; Lambeth and Neish, 2014; Gluschko et al., 2018; Wolf et al., 2020), mitochondria (Afanas’ev et al., 1999; Han et al., 2003; Muller et al., 2004; Murphy, 2009; Brand, 2010; Shoshan-Barmatz et al., 2010; West et al., 2011a,b; Bezawork-Geleta et al., 2017; Zhao et al., 2019; Hadrava Vanova et al., 2020), xanthine oxidase (Nomura et al., 2014; Al-Shehri et al., 2020), peroxisomes (Lismont et al., 2015; Fransen et al., 2017; Shai et al., 2018) and cytochrome P450 oxidases (Omura and Sato, 1962; Zhang et al., 2020) can be responsible for cellular ROS production and it highly depends on the stimulus and the cell type whether a single or multiple ROS sources are activated, for how long this occurs and for what purpose (Banfi et al., 2004; Gavazzi et al., 2006; Bedard and Krause, 2007; Aguirre and Lambeth, 2010; Brand, 2010; Dikalova et al., 2010; Donko et al., 2010; Carnesecchi et al., 2011; Al-Mehdi et al., 2012; Lanciano et al., 2013; Kim et al., 2014; Lambeth and Neish, 2014; Ives et al., 2015; Xu et al., 2017; Herb et al., 2019b; Hernansanz-Agustin et al., 2020; Herb and Schramm, 2021).

O_2_^–^ is the precursor of all cellular ROS (Chavez et al., 2009; Niethammer et al., 2009) and, under physiological pH, cannot diffuse over cell membranes due to its negative charge (Takahashi and Asada, 1983; Shoshan-Barmatz et al., 2010; Cordeiro, 2014). It has a half-life of a couple of seconds or even less (Marklund, 1976; Rapoport et al., 1994; D’Autreaux and Toledano, 2007; Taverne et al., 2013). Despite being not a strong oxidizing substance by itself, O_2_^–^ readily oxidizes iron-sulfur structures of proteins (Liochev and Fridovich, 1999; Imlay, 2003), which can lead to protein malfunction and iron release from proteins. The released iron reacts with H_2_O_2_ to form the highly reactive and toxic OH radical (Liochev and Fridovich, 1999). Therefore, under healthy conditions cells keep O_2_^–^ levels low (∼ 10^–11^-10^–12^ M) by compartmentalization and quick removal of O_2_^–^by superoxide dismutates (D’Autreaux and Toledano, 2007; Kehrer et al., 2010; Wang et al., 2018), which are expressed in all cellular compartments (Melov et al., 1999; Lambeth and Neish, 2014; Wang et al., 2018). A sustained increase in cellular O_2_^–^ levels is associated with damage to cellular structures (Davies, 2016; Pizzino et al., 2017; Sies et al., 2017; Gutteridge and Halliwell, 2018; Su et al., 2019; Juan et al., 2021). However, O_2_^–^ can also contribute to cellular signaling (Chen et al., 2009; Xu et al., 2017; Ren et al., 2021).

O_2_^–^ quickly dismutates to H_2_O_2_, which is more, although not freely, diffusible for cellular membranes (Bienert et al., 2006; Wang et al., 2020; Chauvigne et al., 2021), which questions saturation of the cell with H_2_O_2_ to fulfill signaling functions in compartments, which are not in direct proximity to the ROS source (Beretta et al., 2020; Sies, 2021). Communication between cellular compartments can be achieved by aquaporins, which facilitate a controlled passage of H_2_O_2_ over membranes (Bienert and Chaumont, 2014; Wang et al., 2020). H_2_O_2_ has a longer cellular half-life (∼1 ms) with concentrations of ∼10^–7^ M under cellular homoeostatic conditions (D’Autreaux and Toledano, 2007). Because of these properties, it functions as an important signaling molecule involved in many different cellular processes (Kamata et al., 2005; Tonks, 2005; Rhee, 2006; Marinho et al., 2013; Holmstrom and Finkel, 2014; Romero et al., 2014; Jones et al., 2016; Short et al., 2016; Zhang et al., 2016; Herb et al., 2019b; Sies and Jones, 2020; Chauvigne et al., 2021). H_2_O_2_-mediated signaling is mainly based on the oxidation of cysteine residues of proteins (Chiarugi et al., 2001; Rhee, 2006; Herscovitch et al., 2008; Romero et al., 2014; Jones et al., 2016; Short et al., 2016; Herb et al., 2019b). These cysteine residues have a low pKa, are exposed to the cytosol and deprotonated to thiolate groups (Finkel, 2011; Poole, 2015). An increase to nanomolar concentrations (∼100 nM) of H_2_O_2_ is sufficient to induce reversible oxidation. This can lead to allosteric protein changes that alter the enzymatic function of the target proteins in many ways (Lee et al., 1998; Meng et al., 2002; Kamata et al., 2005; Tonks, 2005). ROS-mediated oxidation can also lead to covalent linkage of cysteine residues by disulfide bonds (Herscovitch et al., 2008; Zhou et al., 2014; Herb et al., 2019b). Since these H_2_O_2_-mediated protein oxidations can be reversed by the antioxidant defense system, they represent important redox switches involved in various cellular processes (Barford, 2004; Holmstrom and Finkel, 2014). Excessive H_2_O_2_ production, however, leads to further oxidation of the oxidized cysteines, which is an irreversible process and results in protein malfunction (Winterbourn and Hampton, 2008).

Growing evidence indicates that the redox status in different cellular compartments varies greatly (Fransen et al., 2017; Beretta et al., 2020; Chauvigne et al., 2021; Sies, 2021; Wang et al., 2021b), is tightly regulated (Nakamura, 2005; Kirkman and Gaetani, 2007; Kelley et al., 2010; Brigelius-Flohe and Maiorino, 2013; Poljsak et al., 2013; Couto et al., 2016; Jones et al., 2016; Chauvigne et al., 2021) and every elevation of ROS levels is controlled by the cell in various ways (Babior, 2002; Bulua et al., 2011; Hoeven et al., 2011; West et al., 2011a; Gluschko et al., 2018; Herb et al., 2019b; Chauvigne et al., 2021). The condition of ROS levels exceeding the capacity of cellular antioxidant defense systems is termed oxidative stress (Niki, 2016; Sies and Jones, 2020). Oxidative stress can be further divided into two subforms: **(1)** Oxidative distress, which represents excessive and prolonged oxidative stress, causes damage to cellular components and results in a number of different pathologies (Cross et al., 1987; Rangasamy et al., 2005; Thimmulappa et al., 2006). Notably, excessive oxidative distress is not always detrimental, if produced at the right place. Exceeded generation of oxidative stress in pathogen-containing phagosomes of phagocytes, for example, is an important factor of antimicrobial immunity (West et al., 2011a; Winterbourn and Kettle, 2013; Gluschko et al., 2018; Herb and Schramm, 2021). **(2)** Oxidative eustress represents a tightly controlled increase in cellular ROS levels (Niki, 2016; Sies and Jones, 2020), which are sufficient to fulfill important cellular processes, but do not induce critical damage to cellular structures (Tai et al., 2009; Finkel, 2011; Nathan and Cunningham-Bussel, 2013; Reczek and Chandel, 2014; Herb et al., 2019b).

Unfortunately, a lot of studies, which show the important role of ROS in various cellular processes, often suggest that ROS are produced in excess, saturate the cell and react randomly with redox-sensitive targets. This is mainly due to experimental setups that might lead to misinterpretation of the location of ROS production in cells.

Many studies use only one type of ROS probe, but do not provide an explanation for the choice, such as specificity for a cellular compartment or a defined type of ROS subspecies. Often probes are used that show neither a specificity for a ROS subspecies nor a defined cellular compartment, which leads to the frequently used terms “intracellular ROS” or “total cellular ROS,” which implicate that ROS once produced are equally distributed in the cell. Common examples for diffusible ROS probes are luminol (Caldefie-Chezet et al., 2002; Pavelkova and Kubala, 2004), 2′,7′-dichlordihydrofluorescein-diacetat (H2DCF-DA) (Ushijima et al., 1997; Hempel et al., 1999; Kim and Xue, 2020; Kim et al., 2021; Wang et al., 2021a) or dihydroethidium (DHE) (Gatliff et al., 2017; Wang and Zou, 2018; Zeller et al., 2021), which are regarded as compartment-specific but in fact they are not (Lundqvist and Dahlgren, 1996; Ushijima et al., 1997; Hempel et al., 1999; Wang and Zou, 2018). There are compartment-specific derivates available for these ROS probes, namely Isoluminol (Lundqvist and Dahlgren, 1996; Dahlgren and Karlsson, 1999; Caldefie-Chezet et al., 2002; Gluschko et al., 2018; Herb et al., 2019b; Wolf et al., 2020), 5-(and −6)-carboxy-2′,7′-dihydrochlorofluorescein-diacetat (5/6-Carboxy-DCF) (Hempel et al., 1999; Mak et al., 2017; Herb et al., 2019b; Wolf et al., 2020) and MitoSOX Red (MitoSOX) (Robinson et al., 2006; Mukhopadhyay et al., 2007) as alternatives, whose combined usage gives a much more confluent picture of the cellular ROS production. The preferable option for most precise ROS measurements concerning compatibility and specificity for ROS subspecies is represented by genetically modified cells, which express the ROS probe of choice in the cellular compartment of choice, like the HyPer family reporters and roGFP2-Orp1 (Belousov et al., 2006; Gutscher et al., 2009; Markvicheva et al., 2011; Bilan et al., 2013; Hernández-Barrera et al., 2013; Wang et al., 2021b). Another precise approach for compartment-specific ROS measurements is the coupling of ROS probes to cargo/particles, which can be engulfed by cells. This technique is especially useful in phagocytes like macrophages, to determine ROS levels in the phagosome (Geng et al., 2015; Ligeon et al., 2021a,b). For further reading on topics regarding ROS detection methods we want to point out to other reviews (Ermakova et al., 2014; Herb and Schramm, 2021).

Also the combined use of only globally working ROS scavengers in combination with ROS probes that detect total cellular ROS can lead to results, which suggest that ROS are present in the whole cell after diffusion from the location of their production. With NAC as most prominent globally working ROS scavenger (Patriarca et al., 2005; Aldini et al., 2018; Ezerina et al., 2018) only the general involvement of ROS in the cellular process of interest can be investigated, but no compartment-specific ROS production can be analyzed. More examples of globally working ROS scavengers are Tempol (4-Hydroxy-Tempo) (dismutation of O_2_^–^ into H_2_O_2_) (Bernardy et al., 2017; Herb et al., 2019b), Tiron (a global O_2_^–^ scavenger) (Krishna et al., 1992; Hein and Kuo, 1998; Manzano et al., 2000), Trolox (globally scavenges OOH and OOR) (Davies et al., 1988; Dugas et al., 2000) and ebselen (effectively removes H_2_O_2_ and ONOO^–^) (Nakamura et al., 2002; Matsushita et al., 2004; Mugesh, 2013). All of the scavengers mentioned above are diffusible (Davies et al., 1988; Krishna et al., 1992; Hein and Kuo, 1998; Haj-Yehia et al., 1999; Dugas et al., 2000; Manzano et al., 2000; Rak et al., 2000; Nakamura et al., 2002; Matsushita et al., 2004; Mugesh, 2013; Herb and Schramm, 2021). Assessment of specific removal of ROS subspecies and therefore their involvement in cellular processes is possible with these substances, but they cannot be used to identify the specific compartment in which the ROS exert their function.

Not only the location of ROS production, but also their various sources and their activation, regulation and termination is of major importance for the understanding of the complex redox maintenance in cells. For the identification of ROS sources it is not always possible to provide genetic evidence with a knock-out system or by siRNA usage. ROS source inhibitors are in these cases an option to block ROS production and analyze possible ROS sources. There are a lot of specific ROS source inhibitors commercially available and the choice is continuously expanded (Murphy, 2009; Wind et al., 2010; Altenhofer et al., 2015; Herb and Schramm, 2021).

For Nox enzymes, as one of the most prominent ROS sources in many cell types, the well-validated general Nox inhibitors VAS2870 (Leusen et al., 1995; ten Freyhaus et al., 2006; Wind et al., 2010; Altenhofer et al., 2012, 2015) or GKT 137831 (Laleu et al., 2010; Sedeek et al., 2010; Gaggini et al., 2011; Aoyama et al., 2012; Strengert et al., 2014; Kim et al., 2021) can be used. Both inhibitors show no intrinsic antioxidant activity and do not inhibit other flavoproteins (Wind et al., 2010; Altenhofer et al., 2012; Teixeira et al., 2017). Both also inhibit Nox-derived ROS production *in vitro* and *in vivo* (Carnesecchi et al., 2009, 2011; Aoyama et al., 2012; Green et al., 2012; Bettaieb et al., 2015; Gorin et al., 2015). In sharp contrast to VAS2870 and GKT 137831, the substances apocynin and DPI are still used and falsely addressed as specific Nox inhibitors in many otherwise convincing and excellent studies (Barbieri et al., 2003; Kiritoshi et al., 2003; Dostert et al., 2008; Choi et al., 2011; Abuaita et al., 2015; Gatliff et al., 2017; Alonso et al., 2019; Fan et al., 2019; Damiano et al., 2020; Geng et al., 2020; Inomata et al., 2020; Prestes et al., 2020; Ahmad et al., 2021; Ligeon et al., 2021b; Martinez et al., 2021 #1039; Troia et al., 2021). Several studies have shown that apocynin directly scavenges ROS due to its antioxidant capacities (Aldieri et al., 2008; Heumuller et al., 2008; Mora-Pale et al., 2009; Wingler et al., 2011; Trevelin et al., 2016), while DPI inhibits flavoproteins in general (O’Donnell et al., 1993; Wind et al., 2010; Altenhofer et al., 2015) including Nox2 (Reis et al., 2020), but also various other targets, such as complex I of the mitochondrial electron transport chain (Bloxham, 1979; Lambeth et al., 2008; Bulua et al., 2011), iNOS (Stuehr et al., 1991; Geyer et al., 1997) or xanthine oxidase (O’Donnell et al., 1993; Wind et al., 2010) as well as calcium transporters (Tazzeo et al., 2009). Since genetic knock-out models with the CRISPR-Cas9 technology (Ledford, 2015; Anzalone et al., 2020; Carlson-Stevermer et al., 2020), either for cell lines, *ex vivo* cells or mice, as well as knock-down *via* siRNA (Han, 2018) are readily available tools for analyzing possible roles of Nox enzymes in cellular processes, the use of apocynin or DPI, especially in combination with diffusible ROS probes, should not be recommended, since it may lead to false interpretations of results regarding Nox enzyme involvement and the location of ROS production.

In mitochondria, the complexes of the ETC not only are essential for energy generation of the cell, but are also ROS production sites (Nohl et al., 2003; Lambeth and Neish, 2014). Inhibition of the complexes for analysis of ROS production might also result in energy deprivation and the energy status of the cell has to be checked every time these inhibitors are used. Typically used inhibitors are rotenone (Stowe and Camara, 2009; Heinz et al., 2017; Scialo et al., 2017), which inhibits complex I and increases ROS production inside the mitochondrial matrix (St-Pierre et al., 2002; Lambert and Brand, 2004; Panov et al., 2005; Stowe and Camara, 2009; Sena et al., 2013) and antimycin A (Murphy, 2009; Bleier and Drose, 2013), which inhibits complex III and increases ROS production into the intermembrane space (IMS) (Chen et al., 2003; Han et al., 2003; Al-Mehdi et al., 2012; Quinlan et al., 2012; Herb et al., 2019a). The most commonly used ROS probe for detection of mitochondrial ROS is MitoSOX, which measures O_2_^–^ exclusively inside the mitochondrial matrix (Robinson et al., 2006; Mukhopadhyay et al., 2007; Ernst et al., 2021). However, since the ETC complexes show compartment-specific differences concerning ROS production (Fridovich, 1997; Murphy, 2009; Brand, 2010; West et al., 2011b; Herb and Schramm, 2021), this probe can only be used to measure ROS production inside mitochondria and, therefore, other cellular compartments should always be analyzed in addition. In healthy, undamaged mitochondria, ROS cannot escape the mitochondrial matrix because of the very effective antioxidative defense system (Roca and Ramakrishnan, 2013; Briston et al., 2017; Hos et al., 2017; Hernansanz-Agustin et al., 2020; Lin et al., 2020; Wang et al., 2020; Zhao et al., 2020). Only after prolonged overproduction or when the structure of the mitochondrial membranes is ruptured, either by opening of the mitochondrial permeability transition pore or direct damage, e.g., by pathogenic toxins, ROS can escape from the matrix into the cytosol (Koterski et al., 2005; Stavru et al., 2011; Roca and Ramakrishnan, 2013; Briston et al., 2017; Hos et al., 2017; Zhang Y. et al., 2019; Zhao et al., 2020). Nevertheless, the general term “mitochondrial ROS” is used in many studies, which often is synonymous for matrix-located mitochondrial ROS production measured by MitoSOX. ROS measurements in other cellular compartments as well as an explanation if and how the mitochondrial ROS escape from the matrix and fulfill their role in the cell, with a few exceptions (Koterski et al., 2005; Zhou et al., 2011; Roca and Ramakrishnan, 2013; Briston et al., 2017; Hos et al., 2017; Herb et al., 2019b; Zhao et al., 2020), are often not provided. Additionally, the usage of inhibitors of the ETC, like rotenone or antimycin A, which have compartment-specific effects on ROS production in combination with diffusible ROS probes can also lead to misinterpretations of the performed ROS measurements. For further reading concerning ROS scavengers and inhibitors, we like to point to other reviews (Wind et al., 2010; Altenhofer et al., 2015; Herb and Schramm, 2021).

## ROS Production: The Dose Makes the Poison

**FIGURE 1:**
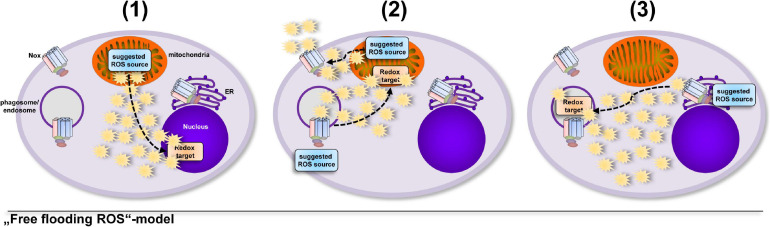
Several studies suggested that ROS are produced in excess, saturate the cell and find their redox-sensitive targets at random. Usage of diffusible ROS probes, globally working ROS scavengers and unspecific inhibitors often place the suggested ROS source at a completely different location than the redox-sensitive target, which might lead to the interpretation that cells “take into account” the damage that ROS can inflict on their way to the target molecule. The importance of ROS in general for various cellular processes was shown by many excellent studies (Bulua et al., 2011; Nazarewicz et al., 2013; Kelly et al., 2015; Garaude et al., 2016; Kim et al., 2017), however, diffusible ROS probes or only one ROS probe are often used to determine ROS production in cells, which might lead to the suggestions, e.g., that **(1)** ROS escape from the mitochondrial matrix and regulate expression and secretion of cytokines (Bulua et al., 2011; Kelly et al., 2015), **(2)** extracellular Nox2-derived ROS modify enzyme activity in the mitochondrial matrix or matrix-derived ROS modulate Nox2 activity (Nazarewicz et al., 2013; Garaude et al., 2016) or **(3)** ROS produced by ER-located Nox4 reach the phagosome for inactivation of phagocytosed parasites (Kim et al., 2017).

**FIGURE 2:**
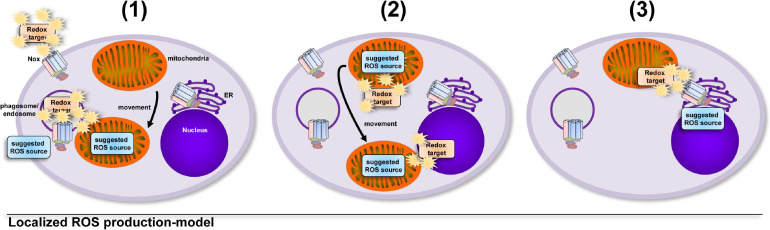
Growing evidence supports the hypothesis that cellular compartments show big differences and tight regulation of their redox status. The induction of ROS production is controlled by the cell in terms of location, source, duration and amount. The localized and timely controlled ROS production in the direct vicinity of the redox-sensitive target reduces the induced damage to cellular components and results in beneficial consequences for the cell, a condition termed as oxidative eustress (Sies, 2021). Examples of localized ROS production are **(1)** the production of antimicrobial ROS by Nox2 (Craig and Slauch, 2009; Gluschko et al., 2018) or mitochondria, which are recruited to pathogen-containing phagosomes (West et al., 2011a; Geng et al., 2015), **(2)** the recruitment of the redox-regulated target to ROS-producing mitochondria for NLRP3 inflammasome activation (Zhou et al., 2011) or the relocation of mitochondria to the nucleus for ROS-mediated nuclear signaling (Al-Mehdi et al., 2012) and **(3)** ROS production by ER-localized Nox4 during formation of mitochondria-associated membranes for regulation of calcium signaling (Beretta et al., 2020).

A model that involves an uncontrolled increase in total cellular ROS levels implies that cells take into account the collateral damage that ROS can inflict while enroute to their redox-sensitive target, that can be at a completely different cellular location (Bulua et al., 2011; Nazarewicz et al., 2013; Kelly et al., 2015; Garaude et al., 2016; Kim et al., 2017; Figure 1). But oxidative distress (Buczynski et al., 2013; Li et al., 2013; Bhattacharyya et al., 2014; Niki, 2016; Sweeney and McAuley, 2016; Yang et al., 2016; Sies and Jones, 2020) is a situation for the healthy cell that has to be avoided. Tightly controlled production of ROS in direct vicinity of a redox-sensitive target (Tai et al., 2009; Finkel, 2011; Nathan and Cunningham-Bussel, 2013; Reczek and Chandel, 2014; Herb et al., 2019b) requires much less ROS production and hence results in much less collateral damage, while fulfilling important cellular functions, i.e., oxidative eustress (Niki, 2016; Sies and Jones, 2020; Figure 2).

Of note, to reach levels at which the ROS can fulfill their important cellular roles, ROS production has to overcome the highly effective antioxidative defense systems of one or more cellular compartments (Nakamura, 2005; Kirkman and Gaetani, 2007; Kelley et al., 2010; Brigelius-Flohe and Maiorino, 2013; Poljsak et al., 2013; Lismont et al., 2015; Couto et al., 2016; Jones et al., 2016; Shai et al., 2018; Chauvigne et al., 2021; Herb and Schramm, 2021). Also H_2_O_2_-detoxifying enzymes, such as glutathione peroxidases or catalase, which can quickly decrease cellular H_2_O_2_ concentrations (Winterbourn and Hampton, 2008) as well as the inactivation of these scavenger enzymes by H_2_O_2_ itself broaden the regulatory potential of cells for selective and localized ROS signaling (Wood et al., 2003; Woo et al., 2010). This further places the model of ROS molecules, which “flood” the whole cell from a single location of ROS production in rather unrealistic light. Nevertheless, the model of ROS as “flooding” the cell is still popular in the scientific community, often referred to as “total cellular ROS levels” (Wang et al., 2013, 2019; Dinakar et al., 2016; Kim and Xue, 2020; Loth et al., 2020; Thorne et al., 2021), “intracellular ROS levels” (Tepel et al., 2000; Bensaad et al., 2009; Choi et al., 2011; Lee et al., 2017; Wang et al., 2019, 2021a; Wei et al., 2019; Zaidieh et al., 2019; Zhang W. et al., 2019; Mendiola et al., 2020; Winitchaikul et al., 2021; Zhong et al., 2021) or simply “ROS levels” (Chen et al., 2012, 2021; Wei et al., 2019; Agarwal and Ganesh, 2020; Kim et al., 2021; Knight et al., 2021; Zeller et al., 2021) in many studies, mainly because of the usage of diffusible ROS probes, which suggest free diffusion of ROS through the cell without regard of the location of ROS production. Mutations, e.g., in cancer cells (Schumacker, 2006; Liou and Storz, 2010; Reczek and Chandel, 2017; Zaidieh et al., 2019; Perillo et al., 2020), pathogenic invasion (West et al., 2011a; Abuaita et al., 2018; Gluschko et al., 2018; Roca et al., 2019) or metabolic disbalance (Li et al., 2016; Mak et al., 2017; Peng et al., 2021) are prominent examples, in which the ROS production of the cell can enter an uncontrolled stage and quickly overcome the antioxidative defense system leading to rapidly increased ROS levels in nearly every compartment of the cell with often detrimental consequences. In this context, cells can be regarded as “overflowing with ROS,” however, in healthy cells a redox balance between all producing and eliminating ROS sources, mediated by the antioxidant defense system is crucial for cellular functioning (Nathan and Cunningham-Bussel, 2013; Deshmukh et al., 2017; Suzuki et al., 2019; Wei et al., 2019; Baird and Yamamoto, 2020; Saito and Kimura, 2021). Importantly, in the extracellular milieu H_2_O_2_ can travel much further than inside the cell and fulfills important signaling (Levine et al., 1994; Sharma et al., 2012; Das and Roychoudhury, 2014; Hervera et al., 2018; Huang et al., 2019; Janku et al., 2019; Deng et al., 2020) and chemotactic functions (Niethammer et al., 2009; Rieger and Sagasti, 2011).

## Concluding Remarks

In recent years, more and more studies supported the model–and highlighted the importance–of localized cellular ROS production in direct vicinity of the redox target (Meinhard and Grill, 2001; Veal et al., 2007; Go and Jones, 2008; Craig and Slauch, 2009; West et al., 2011a; Wink et al., 2011; Zhou et al., 2011; Al-Mehdi et al., 2012; Naviaux, 2012; Allan et al., 2014; Romero et al., 2014; Geng et al., 2015; To et al., 2017; Gluschko et al., 2018; Herb et al., 2019b; Acin-Perez et al., 2020; Beretta et al., 2020; Chanin et al., 2020; Miller et al., 2020; Sies and Jones, 2020; Herb and Schramm, 2021; Ligeon et al., 2021b; Sies, 2021; Wong et al., 2021). Therefore, the model of ROS molecules as “omnipresent and freely diffusing throughout the cell” should always be interpreted carefully in the context of research and highly depends on the proper use of ROS probes, scavengers and inhibitors. In healthy cells, ROS should be considered as molecules, whose production is tightly controlled in terms of stimulus, source, location, duration and amount.

## Data Availability Statement

The original contributions presented in the study are included in the article/supplementary material, further inquiries can be directed to the corresponding author/s.

## Author Contributions

MH and MS: conceptualization. MH: writing–original draft preparation and visualization. MH, AG, and MS: writing–review and editing and funding acquisition. MS: supervision and project administration. All authors have read and agreed to the published version of the manuscript.

## Conflict of Interest

The authors declare that the research was conducted in the absence of any commercial or financial relationships that could be construed as a potential conflict of interest.

## Publisher’s Note

All claims expressed in this article are solely those of the authors and do not necessarily represent those of their affiliated organizations, or those of the publisher, the editors and the reviewers. Any product that may be evaluated in this article, or claim that may be made by its manufacturer, is not guaranteed or endorsed by the publisher.
